# Use of COVID-19 Vaccines After Reports of Adverse Events Among Adult Recipients of Janssen (Johnson & Johnson) and mRNA COVID-19 Vaccines (Pfizer-BioNTech and Moderna): Update from the Advisory Committee on Immunization Practices — United States, July 2021

**DOI:** 10.15585/mmwr.mm7032e4

**Published:** 2021-08-13

**Authors:** Hannah G. Rosenblum, Stephen C. Hadler, Danielle Moulia, Tom T. Shimabukuro, John R. Su, Naomi K. Tepper, Kevin C. Ess, Emily Jane Woo, Adamma Mba-Jonas, Meghna Alimchandani, Narayan Nair, Nicola P. Klein, Kayla E. Hanson, Lauri E. Markowitz, Melinda Wharton, Veronica V. McNally, José R. Romero, H. Keipp Talbot, Grace M. Lee, Matthew F. Daley, Sarah A. Mbaeyi, Sara E. Oliver

**Affiliations:** ^1^CDC COVID-19 Response Team; ^2^Epidemic Intelligence Service, CDC; ^3^Vanderbilt University School of Medicine, Nashville, Tennessee; ^4^Food and Drug Administration, Silver Spring, Maryland; ^5^Division of Research, Kaiser Permanente Northern California, Oakland, California; ^6^Marshfield Clinic Research Institute, Marshfield, Wisconsin; ^7^Franny Strong Foundation, West Bloomfield, Michigan; ^8^Arkansas Department of Health; ^9^Stanford University School of Medicine, Stanford, California; ^10^Institute for Health Research, Kaiser Permanente Colorado, Denver, Colorado.

In December 2020, the Food and Drug Administration (FDA) issued Emergency Use Authorizations (EUAs) for Pfizer-BioNTech and Moderna COVID-19 vaccines, and in February 2021, FDA issued an EUA for the Janssen (Johnson & Johnson) COVID-19 vaccine. After each EUA, the Advisory Committee on Immunization Practices (ACIP) issued interim recommendations for vaccine use; currently Pfizer-BioNTech is authorized and recommended for persons aged ≥12 years and Moderna and Janssen for persons aged ≥18 years (*1*–*3*). Both Pfizer-BioNTech and Moderna vaccines, administered as 2-dose series, are mRNA-based COVID-19 vaccines, whereas the Janssen COVID-19 vaccine, administered as a single dose, is a recombinant replication-incompetent adenovirus-vector vaccine. As of July 22, 2021, 187 million persons in the United States had received at least 1 dose of COVID-19 vaccine (*4*); close monitoring of safety surveillance has demonstrated that serious adverse events after COVID-19 vaccination are rare (*5*,*6*). Three medical conditions have been reported in temporal association with receipt of COVID-19 vaccines. Two of these (thrombosis with thrombocytopenia syndrome [TTS], a rare syndrome characterized by venous or arterial thrombosis and thrombocytopenia, and Guillain-Barré syndrome [GBS], a rare autoimmune neurologic disorder characterized by ascending weakness and paralysis) have been reported after Janssen COVID-19 vaccination. One (myocarditis, cardiac inflammation) has been reported after Pfizer-BioNTech COVID-19 vaccination or Moderna COVID-19 vaccination, particularly after the second dose; these were reviewed together and will hereafter be referred to as mRNA COVID-19 vaccination. ACIP has met three times to review the data associated with these reports of serious adverse events and has comprehensively assessed the benefits and risks associated with receipt of these vaccines. During the most recent meeting in July 2021, ACIP determined that, overall, the benefits of COVID-19 vaccination in preventing COVID-19 morbidity and mortality outweigh the risks for these rare serious adverse events in adults aged ≥18 years; this balance of benefits and risks varied by age and sex. ACIP continues to recommend COVID-19 vaccination in all persons aged ≥12 years. CDC and FDA continue to closely monitor reports of serious adverse events and will present any additional data to ACIP for consideration. Information regarding risks and how they vary by age and sex and type of vaccine should be disseminated to providers, vaccine recipients, and the public.

Since June 2020, ACIP has convened 16 public meetings to review data on COVID-19 epidemiology and use of COVID-19 vaccines, most recently on July 22, 2021. The ACIP COVID-19 Vaccines Work Group, comprising experts in infectious diseases, vaccinology, vaccine safety, public health, and ethics, has held weekly meetings since April 2020 to review COVID-19 surveillance data, evidence for vaccine efficacy and safety, and implementation considerations for COVID-19 vaccination programs.

ACIP met to review reports of TTS after Janssen COVID-19 vaccination in April 2021; the committee met again in June 2021 to review reports of myocarditis after mRNA COVID-19 vaccination, particularly after the second dose. At both meetings, ACIP reviewed the individual- and population-level benefits and risks for vaccination and concluded that the benefits of vaccination for individual persons and at the population-level outweigh the risks; details of the findings have been described previously (*7*,*8*). FDA added information about these serious adverse events to the EUA fact sheets*; CDC updated patient and clinician education and communication materials,^†^ and federal agencies continue to closely monitor reports of these serious adverse events.

On July 12, 2021, FDA issued a warning and updated EUA fact sheets after reports of a more than expected number of GBS cases to the Vaccine Adverse Events Reporting System (VAERS) after Janssen COVID-19 vaccination. GBS is a rare neurologic disorder characterized by acute or subacute onset of weakness in limbs or cranial nerve–innervated muscles and by laboratory findings of increased cerebrospinal fluid protein with normal numbers of cells; the clinical presentation and severity vary (*9*). GBS occurs more commonly in males than in females, and incidence increases with age; 3,000–6,000 GBS cases are reported annually in the United States.^§^ Patients might require intensive care unit (ICU) admission and ventilator support; although most patients recover, GBS can result in permanent paralysis or death (*10*).

After the reports of GBS cases after Janssen COVID-19 vaccination, the Work Group met to review clinical trial and postauthorization safety data for GBS. To comprehensively evaluate the benefits and risks associated with COVID-19 vaccination, in addition to reviewing a benefit-risk assessment of GBS after Janssen COVID-19 vaccination, the Work Group also updated benefit-risk assessments of TTS cases after Janssen COVID-19 vaccination and of myocarditis cases after mRNA COVID-19 vaccination in adults aged ≥18 years. The ACIP COVID-19 Vaccines Safety Technical (VaST) Work Group,^¶^ comprising independent vaccine safety expert consultants, performed concomitant review of the adverse events information.

On July 22, 2021, ACIP met to review currently available evidence of risks associated with COVID-19 vaccination. The findings from VaST and ACIP COVID-19 Vaccine Work Group assessments, including a summary of the data reviewed, were presented to ACIP during this meeting. ACIP’s comprehensive assessment included risks for GBS and TTS after Janssen COVID-19 vaccination and myocarditis after mRNA COVID-19 vaccination in persons aged ≥18 years. To date, there has been no increased risk detected for GBS or TTS after mRNA COVID-19 vaccination, and there has been no increased risk detected for myocarditis after Janssen COVID-19 vaccination. Persons aged <18 years were not included in this assessment because a benefit-risk assessment for persons aged 12–29 years was recently presented to ACIP in June 2021**; ongoing safety monitoring continues and can be included in future updates to ACIP (*8*).

To assess the benefit-risk balance of COVID-19 vaccination in adults, ACIP reviewed an assessment comparing the benefits of vaccination (numbers of COVID-19 cases and severe disease outcomes prevented) to the risks (numbers of cases of GBS, TTS, and myocarditis), using methods similar to those described previously.^††^ Specifically, the benefits per million vaccine doses administered (i.e., the benefits of being fully vaccinated^§§^ in accordance with the FDA EUA) were assessed, including 1) COVID-19 cases prevented, based on rates during the week of June 13–19, 2021^¶¶^; 2) COVID-19 hospitalizations prevented, based on rates during the week of June 19, 2021***; and 3) COVID-19 ICU admissions and deaths prevented, based on the proportion of hospitalized patients who were admitted to an ICU or who died.^†††^

The risks assessed for the Janssen COVID-19 vaccination were 1) the number of GBS patients reported to VAERS that occurred within 42 days of Janssen COVID-19 vaccination per million doses administered through June 30, 2021, and 2) the number of patients with TTS reported to VAERS that occurred after Janssen COVID-19 vaccination per million doses through July 8, 2021. The risks for mRNA COVID-19 vaccination were assessed as the number of patients reported to VAERS with myocarditis after receipt of dose 2 of an mRNA COVID-19 vaccine per million doses. Each benefit-risk assessment was stratified by age group (18–29, 30–49, 50–64 and ≥65 years) and sex. The Janssen COVID-19 vaccine analysis assumed 90% vaccine effectiveness^§§§^ in preventing severe outcomes and 66% vaccine effectiveness in preventing COVID-19 cases for a 120-day period. The mRNA COVID-19 vaccine analysis assumed 95% vaccine effectiveness^¶¶¶^ in preventing severe outcomes and in preventing COVID-19 cases for a 120-day-period. The 120-day period was selected because inputs pertaining to community transmission have increased uncertainty beyond this period, particularly with regard to virus variants in circulation.**** Using GBS, TTS, and myocarditis cases reported to VAERS with age and sex data available, crude reporting rates^††††^ per million vaccine doses administered were calculated, overall and among subgroups, by sex and age using national COVID-19 vaccine administration data. GBS rates from the Vaccine Safety Datalink (VSD),^§§§§^ based on cases confirmed by medical record review, were also presented to and reviewed by ACIP.

As of June 30, 2021, approximately 12.6 million doses of Janssen COVID-19 vaccine had been administered in the United States to persons aged ≥18 years. Within VAERS,^¶¶¶¶^ 100 reports of GBS after Janssen COVID-19 vaccination were received during February 27–June 30, 2021. The median patient age was 57 years (range = 24–76); 61 (61%) were males, and the median interval from vaccination to symptom onset was 13 days (range = 0–75 days). Ninety-five (95%) patients experiencing GBS were hospitalized, and 10 (10%) were admitted to an ICU. Ninety-eight (98%) of these patients had disease onset within 42 days of vaccination. As of the most recent follow-up,***** one patient had died. The GBS reporting rate was 7.8 cases per million Janssen COVID-19 vaccine doses administered. Among subgroups by sex and age, the reporting rate to VAERS was highest among males aged 50–64 years, with 15.6 cases per million Janssen COVID-19 vaccine doses administered (Table 1). VSD has not identified a signal^†††††^ for GBS after Janssen COVID-19 vaccination. However, based on medical record–confirmed GBS cases reported during the 21 days^§§§§§^ after receipt of Janssen COVID-19 vaccine, the unadjusted GBS rate in VSD was 20.2 per million doses administered (95% confidence interval = 8.1–41.7).^¶¶¶¶¶^

**TABLE 1:** Number of Guillain-Barré syndrome cases* reported to the Vaccine Adverse Events Reporting System within 42 days after Janssen (Johnson & Johnson) COVID-19 vaccination, total Janssen doses administered, and reporting rate per million doses administered, by sex and age group — United States, February –June 2021

Sex/Age group, yrs	GBS cases^†^	No. of doses administered	GBS cases per million vaccine doses administered
**Females**			
18–29	1	1,037,996	1.0
30–49	13	1,957,663	6.6
50–64	14	1,888,715	7.4
≥65	9	1,037,996	8.7
**Total females**	**37**	**5,922,370**	6.2
**Males**			
18–29	3	1,258,963	2.4
30–49	18	2,407,430	7.5
50–64	33	2,115,411	15.6
≥65	7	932,764	7.5
**Total males**	**61**	**6,714,598**	**9.1**
**Total**	**98**	**12,636,938**	**7.8**

**Abbreviations:** GBS = Guillain-Barré syndrome; VAERS = Vaccine Adverse Events Reporting System.

* Unconfirmed cases reported to VAERS.

^†^ 100 cases total were reported to VAERS during this period; the 98 displayed here occurred within 42 days of vaccination and had age and sex information available.

Through July 8, 2021, 38 cases of TTS within 15 days of vaccination and reported to VAERS met the case definition.****** These 38 reports were confirmed by physician reviewers at CDC and FDA and reviewed with the Clinical Immunization Safety Assessment Project Investigators, who include hematologists. Four of these patients died. The overall TTS reporting rate was 3.0 cases per million doses administered as of July 8, 2021.^††††††^ Among subgroups by sex and age, the reporting rate was highest among females aged 30–49 years (8.8 TTS cases per million Janssen COVID-19 vaccine doses administered).

As of June 30, 2021, approximately 141 million second mRNA COVID-19 vaccine doses had been administered in the United States to persons aged ≥18 years. Within VAERS, 497 reports of myocarditis after the second mRNA COVID-19 vaccine dose were received for persons aged ≥18 years. The reporting rate of myocarditis overall among adults was 3.5 cases per million second doses of mRNA COVID-19 vaccine administered. In subgroup analyses by age and sex, the reporting rate was highest among males aged 18–29 years (24.3 cases per million mRNA COVID-19 vaccine second doses administered). Reports of cases in persons aged 18–29 years were individually reviewed and confirmed to meet case definitions, whereas reports of cases in persons aged ≥30 years were received and processed^§§§§§§^ but not individually reviewed. There were no confirmed myocarditis-associated deaths.

The estimated benefits (prevention of COVID-19 disease and associated hospitalizations, ICU admissions, and deaths) outweighed the risks (expected cases of GBS, TTS, and myocarditis after vaccination) in all persons aged ≥18 years included in this analysis (Table 2). For example, per million doses of Janssen COVID-19 vaccine administered to males aged 50–64 years, 1,800 hospitalizations, 480 ICU admissions, and 140 deaths attributable to COVID-19 could be prevented, compared with 14–17 GBS cases and 1–2 TTS cases after Janssen COVID-19 vaccination. However, the balance of benefits and risks varied by age and sex because cases of each serious adverse event were primarily identified in specific subgroups of age and sex (primarily males aged 50–64 years for GBS; females aged 30–49 years for TTS; and males aged 18–29 years for myocarditis).

**TABLE 2:** Estimated COVID-19 outcomes prevented during 120 days after 1-dose Janssen (Johnson & Johnson) COVID-19 vaccination and 2-dose mRNA (Pfizer-BioNTech or Moderna) COVID-19 vaccination, number of Guillain-Barré syndrome and thrombosis with thrombocytopenia syndrome cases expected per million Janssen vaccine doses administered, and number of myocarditis cases expected per million second mRNA vaccine doses administered, by sex and age group — United States, 2021*

Vaccine	Benefits: COVID-19 outcomes prevented				Harms: adverse events^†^	
Sex/Age group, yrs	Cases	Hospitalizations	ICU admissions	Deaths	GBS	TTS
**Janssen (Johnson & Johnson) COVID-19 vaccine^§^**						
**Females**						
18–29	8,900	700	50	5	1	4–5
30–49	10,100	900	140	20	6–7	8–10
50–64	12,100	1,600	350	120	7–8	3–4
≥65	29,000	5,900	1,250	840	8–10	0
**Males**						
18–29	6,600	300	60	3	2	2–3
30–49	7,600	650	150	25	7–8	1–2
50–64	10,100	1,800	480	140	14–17	1–2
≥65	36,600	11,800	3,300	2,300	7–8	0
**mRNA (Pfizer-BioNTech or Moderna) COVID-19 vaccine** ^¶^					**Myocarditis**	
**Females**						
18–29	12,800	750	50	5	3–4	
30–49	14,600	950	140	20	1–2	
50–64	17,500	1,700	375	125	1	
≥65	32,000	6,200	1,300	900	<1	
**Males**						
18–29	9,600	300	60	3	22–27	
30–49	11,000	700	160	25	5–6	
50–64	14,700	1,900	500	150	1	
≥65	52,700	12,500	3,500	2,400	1	

**Abbreviations:** GBS = Guillain-Barré syndrome; ICU = intensive care unit; TTS = thrombosis with thrombocytopenia syndrome.

* Benefits and harms were calculated using case incidence and hospitalization data for the week ending June 19, 2021, and for harms using cases through June 30 (GBS and myocarditis) and through July 8 (TTS), projected for a 120-day period using methods described here: https://www.cdc.gov/vaccines/covid-19/info-by-product/janssen/risk-benefit-analysis.html

^†^ Estimates for adverse events are based on an estimated risk of cases per million doses administered with a +/- 10% range.

^§^ Benefits and harms calculated per million doses of Janssen vaccine administered.

^¶^ Benefits and harms calculated per million second doses of mRNA (Pfizer-BioNTech and Moderna) vaccine administered.

ACIP also reviewed population-level considerations, including that COVID-19 cases are rising in the United States, particularly with the predominance of the highly transmissible B.1.617.2 (Delta) variant. More than one half (61%) of U.S. adults aged ≥18 years are fully vaccinated (*4*); however, coverage is lower in some geographic regions. According to a jurisdictional survey conducted on July 16, 2021, most vaccination sites offer more than one type of vaccine and report that Janssen vaccine is used in a variety of populations and settings.^¶¶¶¶¶¶^

Based on a comprehensive review of existing data, in the context of ongoing transmission of SARS-CoV-2, the virus that causes COVID-19, in the United States as of July 2021, the ACIP concluded that 1) the benefits of vaccinating all recommended age groups with either the Janssen COVID-19 vaccine or mRNA COVID-19 vaccine outweigh the risks for vaccination, including the risks for GBS and TTS after Janssen COVID-19 vaccination, or myocarditis after mRNA COVID-19 vaccination; 2) continuing safety monitoring of serious adverse events after COVID-19 vaccination is critical; and 3) providers and the public should be informed about these potential harms and the use of COVID-19 vaccines. The analysis did not include potential benefits of preventing post–COVID-19 conditions, or likely ongoing benefits beyond the 120-day period; for these reasons, the benefits of COVID-19 vaccination are underestimated.

ACIP members discussed concerns about the clinical severity of the rare risk for GBS and TTS. In addition, they noted the importance of providing options for the type of COVID-19 vaccines offered, especially in the context of the current COVID-19 epidemiology and current vaccine coverage in the United States. ACIP emphasized the importance of informing vaccination providers, and all persons receiving COVID-19 vaccines about the benefits and risks, including the risks after Janssen COVID-19 vaccination for GBS, particularly in males aged 50–64 years, and for TTS among females aged 30–49; and the risk for myocarditis after mRNA COVID-19 vaccination, particularly in males aged 18–29 years. CDC has provided guidance regarding evaluation and management of GBS, TTS, and myocarditis.******* In addition to information about TTS, FDA has added information to the Janssen COVID-19 vaccine EUA and fact sheets regarding GBS cases that have been reported among vaccine recipients. The vaccine product-specific EUA fact sheet should be provided to all persons before vaccination with any authorized COVID-19 vaccine.

CDC has updated patient education and communication materials^†††††††^ reflecting this information; these are important to ensure that vaccine recipients are aware of risks and that they should seek care if they experience concerning symptoms. Persons should be educated about their individual benefits and risks associated with COVID-19 vaccination, and when feasible, provided a choice about which type of COVID-19 vaccine to receive.

Based on ACIP’s conclusion regarding the benefit-risk assessment on July 22, 2021, vaccination with any of the available COVID-19 vaccines licensed under the FDA EUAs continues to be recommended for all persons aged ≥18 years. With the Delta variant, this is more urgent than ever. In addition, the Pfizer-BioNTech COVID-19 vaccine continues to be recommended for persons aged ≥12 years.

CDC and FDA will continue to closely monitor reports of serious adverse events and will present any additional data to ACIP for consideration. The benefit-risk analyses for COVID-19 vaccines can be updated to reflect changes in epidemiology of the COVID-19 pandemic and additional information on the risk for serious adverse events after vaccination. ACIP recommendation for use of all COVID-19 vaccines under an EUA are interim and will be updated as additional information becomes available.

## Reporting of Vaccine Adverse Events

FDA requires that vaccine providers report to VAERS vaccination administration errors, serious adverse events,^§§§§§§§^ cases of multisystem inflammatory syndrome, and cases of COVID-19 that result in hospitalization or death after administration of a COVID-19 vaccine under an EUA. CDC also encourages reporting of any additional clinically significant adverse event, even if it is not clear whether a vaccination caused the event. Information on how to submit a report to VAERS is available at https://vaers.hhs.gov/index.html or 1-800-822-7967. In addition, CDC has developed a voluntary smartphone-based online tool (v-safe) that uses text messaging and online surveys to provide near real-time health check-ins after receipt of a COVID-19 vaccine. In cases of v-safe reports that include possible medically attended health events, CDC’s v-safe call center follows up with the vaccine recipient to collect additional information for completion of a VAERS report. Information on v-safe is available at https://www.cdc.gov/vsafe.

**Summary**

**What is already known about this topic?**

Rare serious adverse events have been reported after COVID-19 vaccination, including Guillain-Barré syndrome (GBS) and thrombosis with thrombocytopenia syndrome (TTS) after Janssen COVID-19 vaccination and myocarditis after mRNA (Pfizer-BioNTech and Moderna) COVID-19 vaccination.

**What is added by this report?**

On July 22, 2021, the Advisory Committee on Immunization Practices reviewed updated benefit-risk analyses after Janssen and mRNA COVID-19 vaccination and concluded that the benefits outweigh the risks for rare serious adverse events after COVID-19 vaccination.

**What are the implications for public health practice?**

Continued COVID-19 vaccination will prevent COVID-19 morbidity and mortality far exceeding GBS, TTS, and myocarditis cases expected. Information about rare adverse events should be disseminated to providers, vaccine recipients, and the public.

## Footnotes

* https://www.fda.gov/media/146304/download; https://www.fda.gov/media/146305/download

†
https://www.cdc.gov/coronavirus/2019-ncov/vaccines/index.html

§
https://www.cdc.gov/vaccinesafety/concerns/guillain-barre-syndrome.html

¶
https://www.cdc.gov/vaccines/acip/work-groups-vast/index.html

**
https://www.cdc.gov/vaccines/acip/meetings/slides-2021-06.html

††
https://www.cdc.gov/vaccines/covid-19/info-by-product/janssen/risk-benefit-analysis.html

§§
https://www.cdc.gov/coronavirus/2019-ncov/vaccines/fully-vaccinated.html

¶¶ https://covid.cdc.gov/covid-data-tracker/#demographicsovertime. Data were used for the most recent week not subject to reporting delays before the ACIP meeting.

*** https://gis.cdc.gov/grasp/COVIDNet/COVID19_3.html. Data were used for the most recent week not subject to reporting delays before the ACIP meeting.

†††
https://gis.cdc.gov/grasp/COVIDNet/COVID19_5.html

§§§ Vaccine effectiveness for Janssen COVID-19 vaccine based on data from phase 3 clinical trial.

¶¶¶ Vaccine effectiveness for Pfizer-BioNTech and Moderna COVID-19 vaccines based on data from phase 3 clinical trials.

****
https://covid.cdc.gov/covid-data-tracker/#variant-proportions

†††† GBS reporting rates were calculated using unconfirmed cases. TTS reporting rates were calculated using confirmed cases. Myocarditis reporting rates included confirmed cases for aged 18–29 years and unconfirmed cases for aged ≥30 years.

§§§§
https://www.cdc.gov/vaccinesafety/ensuringsafety/monitoring/vsd/index.html

¶¶¶¶
https://vaers.hhs.gov/index.html

*****
https://www.cdc.gov/vaccines/acip/meetings/downloads/slides-2021-07/02-COVID-Alimchandani-508.pdf

††††† The term signal in VSD refers to a prespecified statistical signal signifying risk.

§§§§§ Note that VSD used a risk length of 21 days, compared with VAERS, which used 42 days.

¶¶¶¶¶
https://www.cdc.gov/vaccines/acip/meetings/downloads/slides-2021-07/03-COVID-Klein-508.pdf

******
https://brightoncollaboration.us/thrombosis-with-thrombocytopenia-syndrome-interim-case-definition/

†††††† Calculations of reporting rates for TTS used denominators of Janssen doses administered through July 8, 2021.

§§§§§§ Processed VAERS reports are those that have been coded using MedDRA, have been deduplicated, and have undergone standard quality assurance and quality control review.

¶¶¶¶¶¶
https://www.cdc.gov/vaccines/acip/meetings/downloads/slides-2021-07/06-COVID-Mbaeyi-508.pdf

*******
https://www.cdc.gov/vaccines/covid-19/clinical-considerations/covid-19-vaccines-us.html

†††††††
https://www.cdc.gov/coronavirus/2019-ncov/vaccines/safety/adverse-events.html

§§§§§§§
https://vaers.hhs.gov/reportevent.html
